# Characterization of Low-Level Glyphosate Tolerance in an *Abutilon theophrasti* Population from a Soybean Field

**DOI:** 10.3390/plants15203186

**Published:** 2026-10-17

**Authors:** Jiabo Dong, Lijun Wang, Xiaofei Ding, Qingkang Wei, Shenao Hu, Xinhui Xue, Lichun Huang, Hong Ma, Zhaofeng Huang

**Affiliations:** 1 College of Plant Protection, Northeast Agricultural University, Harbin 150030, China; 18945473910@163.com (J.D.);; 2 State Key Laboratory for Biology of Plant Diseases and Insect Pests, Institute of Plant Protection (IPP), Chinese Academy of Agricultural Sciences (CAAS), Beijing 100193, China; nmgwang2015@163.com (L.W.);; 3 Inner Mongolia Innovation Center of Biological Breeding Technology, Hinggan League Institute of Agricultural and Husbandry Sciences, Ulan Hot 137400, China

**Keywords:** *Abutilon theophrasti*, glyphosate, low-level tolerance, *EPSPS* activity, non-target-site mechanism, weed management

## Abstract

This study investigated the tolerance level and mechanism of a glyphosate-tolerant *Abutilon theophrasti* population (T2784) collected from a glyphosate-resistant soybean field. Whole-plant dose–response, *EPSPS* sequencing, gene expression analyses, EPSPS activity assays, glyphosate absorption and metabolism tests, and a malathion inhibition assay were performed. The GR_50_ values were 953.0 g a.i. ha^−1^ for T2784 and 415.3 g a.i. ha^−1^ for the susceptible S2857 population, yielding a tolerance index of 2.29. No amino acid substitutions were detected in the amplified *EPSPS* region. However, *EPSPS* genomic copy number was not assessed, and therefore *EPSPS* amplification cannot be excluded. No significant differences in *EPSPS* expressions were observed between the two populations. T2784 showed significantly higher constitutive EPSPS activity and a 2.66-fold higher IC_50_ for glyphosate inhibition than S2857. No differences in glyphosate absorption or in the estimated disappearance of recoverable parent glyphosate were detected. Because specific glyphosate metabolites were not quantified, the estimated metabolism values represent operational estimates based on parent-compound disappearance rather than direct measurements of glyphosate biotransformation. Malathion pretreatment did not significantly alter GR_50_ values in either population. The lower glyphosate sensitivity of T2784 was associated with higher EPSPS activity and a higher inhibition IC_50_ measured in vitro. However, the causal basis of the whole-plant response remains unresolved. Further studies are needed to clarify the biochemical basis of elevated EPSPS activity and to directly characterize whole-plant glyphosate translocation and metabolic products. This study provides a basis for further investigating non-target-site processes and supports the use of integrated weed-management strategies in glyphosate-resistant soybean production systems.

## 1. Introduction

Soybean is one of the world’s most important oilseed and protein crops and plays a major role in meeting global food and nutritional needs [1]. Despite sustained policy support, domestic soybean production in China continues to face significant challenges, failing to form an effective substitute for massive imports. Low per-unit yields, high production costs, low oil content, and poor seed uniformity collectively contribute to a persistent dilemma of “increased production without enhanced profitability” [2]. Where glyphosate is used, differences in glyphosate sensitivity among *A. theophrasti* populations may affect control. However, the traits associated with low-level differences in glyphosate response in this species remain insufficiently characterized. Glyphosate-resistant transgenic soybeans were first commercialized for large-scale cultivation in 1996 in the global area [3], and China formally initiated a pilot program for the commercial production of transgenic maize and soybean in 2021 [4]. Owing to their advantages in herbicide tolerance, high yield potential, and reduced production costs, glyphosate-resistant transgenic soybeans have been rapidly adopted worldwide, with the global planting area continuing to expand annually [5,6].

Among the major biotic constraints to soybean production, *Abutilon theophrasti* Medik, a competitive annual broadleaf weed native to East and South Asia, Europe, and North America, is now widely distributed across China, India, Japan, Europe, and the Americas [7,8]. By competing for critical resources such as light, water, and nutrients, velvetleaf can cause soybean yield losses as high as 50% [9,10,11]. Moreover, a single plant can produce up to 17,000 seeds, substantially limiting soybean yield potential [12,13]. This problem is particularly pronounced in regions reliant on glyphosate-resistant soybean cultivars. Since the widespread adoption of transgenic soybean in the late 1990s, glyphosate has revolutionized weed management; however, it has also inadvertently selected for glyphosate-tolerant velvetleaf populations, thereby complicating control efforts and causing billions of dollars in economic losses annually worldwide [14]. The widespread occurrence of glyphosate-tolerant weeds in global agricultural systems thus poses a major challenge to effective weed management and sustainable farming practices.

Glyphosate (N-(phosphonomethyl)glycine) is a widely used, non-selective organophosphorus herbicide. Its primary mode of action involves the inhibition of 5-enolpyruvylshikimate-3-phosphate synthase (EPSPS), a key enzyme in the shikimate pathway, thereby blocking the biosynthesis of the aromatic amino acids phenylalanine, tyrosine, and tryptophan, ultimately leading to growth inhibition and eventual plant death [15]. The development and commercialization of glyphosate-resistant transgenic crops began with the successful engineering of glyphosate-tolerant tobacco plants and have since become a sustained focus of plant biotechnology research, culminating in the development of resistant varieties in major commodity crops such as soybean, maize, and cotton. Recent studies further suggest that glyphosate may exert additional herbicidal effects through the induction of oxidative stress and disruption of phytohormone homeostasis, particularly auxin and ethylene signaling [16]. Furthermore, mutations or amplification of the *EPSPS* gene in certain resistant weed populations can compromise glyphosate efficacy [17]. Collectively, the mechanistic understanding of glyphosate action not only provides a theoretical foundation for the development of novel herbicides but also raises critical concerns regarding environmental persistence and resistance management [18].

The mechanisms underlying weed tolerance to glyphosate are closely related to those governing resistance, yet the former remain far less comprehensively studied than the latter. To date, glyphosate resistance in weeds has been attributed to two major categories: target-site and non-target-site mechanisms [19]. Target-site resistance primarily involves mutations in the *EPSPS* gene that result in amino acid substitutions, thereby reducing glyphosate binding affinity to the target enzyme [20], and enhanced transcriptional expression [21]. Non-target-site mechanisms, in contrast, include morphological and physiological traits that reduce glyphosate uptake, as well as enhanced metabolic detoxification of the herbicide within plant tissues [22].

This study compares the glyphosate responses of one *A. theophrasti* population collected from a soybean field (T2784) and one susceptible reference population (S2857). We assessed several candidate traits associated with their different responses; the study was not designed to identify a general mechanism for the species. Specifically, we aim to (1) determine the sensitivity levels of different *A. theophrasti* populations to glyphosate using whole-plant dose–response bioassays; (2) amplify and compare the sequenced *EPSPS* region between the two populations; (3) analyze *EPSPS* gene relative expression levels, and corresponding enzyme activity to investigate target-site tolerance mechanisms; (4) quantify glyphosate absorption and estimate the disappearance of recoverable parent glyphosate from the treated leaf using ultra-high-performance liquid chromatography–tandem mass spectrometry; and (5) evaluate the possible contribution of CYP450-dependent activity using the metabolic inhibitor malathion. By integrating whole-plant, molecular, biochemical, and analytical approaches, this study aimed to characterize the basis of the differential glyphosate response between the two populations and provide information for the sustainable management of *A. theophrasti* in glyphosate-resistant soybean production systems.

## 2. Results

### 2.1. Whole-Plant Glyphosate Sensitivity Assay

The whole-plant dose–response assay showed that the T2784 population exhibited measurable tolerance to glyphosate. Following treatment with the recommended field dose of 1125 g a.i. ha^−1^, at 21 days after treatment, all plants in the susceptible S2857 population had died, whereas 83.3% of the T2784 plants survived.

**Figure 1:**
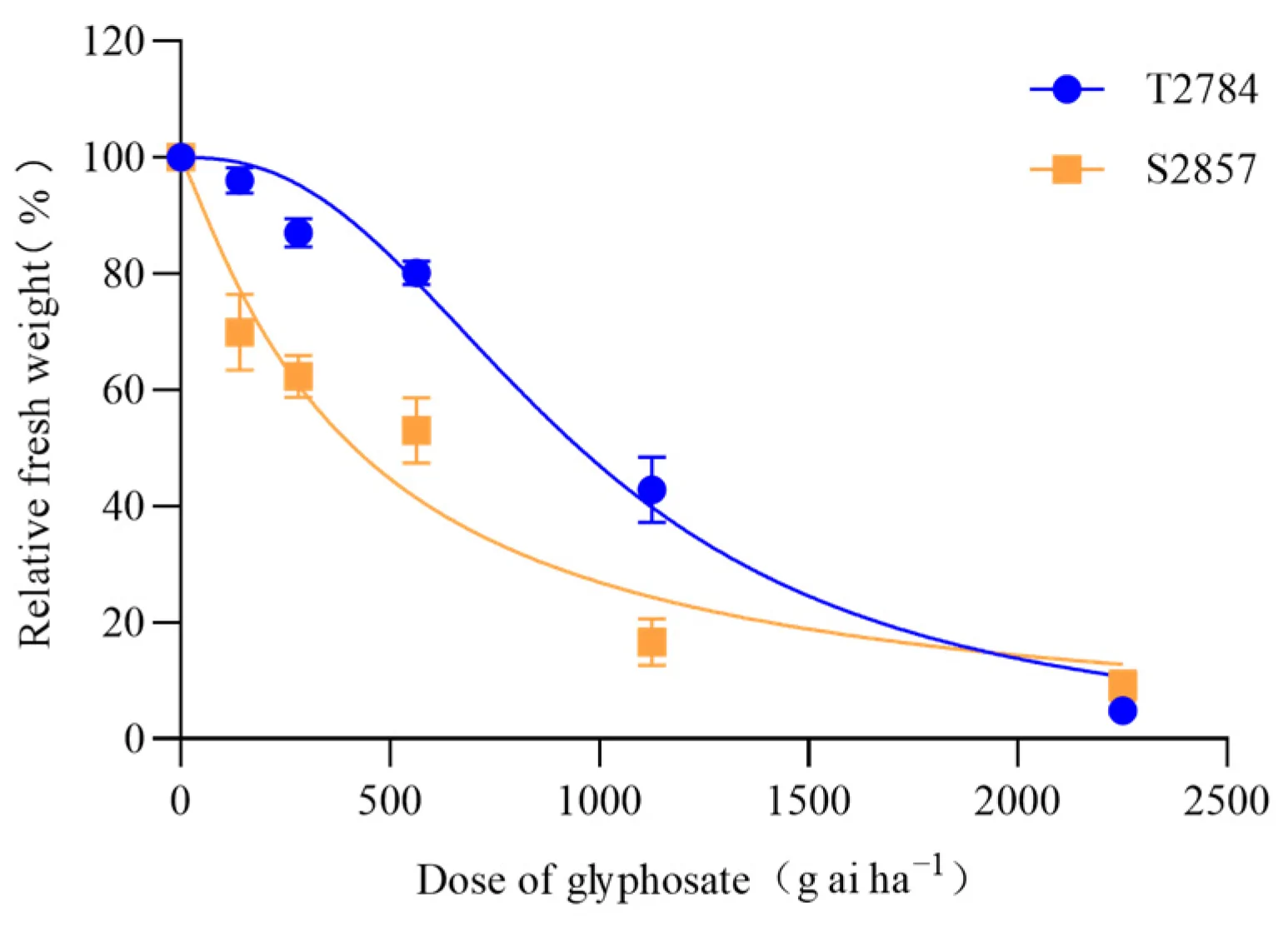
Dose–response curves with glyphosate treatment in the S2857 and T2784 *Abutilon theophrasti* populations.

The estimated GR_50_ was 953.0 g a.i. ha^−1^ for T2784, with a 95% confidence interval of [872.9–1038], and 415.3 g a.i. ha^−1^ for S2857, with a 95% confidence interval of [336.3–505.3]. The tolerance index was 2.29, indicating a low level of glyphosate tolerance (Table 1; Figure 1). We use the term “low-level glyphosate tolerance” descriptively to indicate the lower sensitivity of T2784 under the present experimental conditions. This ratio alone does not establish a formally defined case of evolved glyphosate resistance. Here, “low-level glyphosate tolerance” describes the lower sensitivity of T2784 relative to S2857 under the present experimental conditions. The ratio alone does not establish a formally characterized case of evolved glyphosate resistance.

### 2.2. EPSPS Gene Sequence Analysis

**Figure 2:**
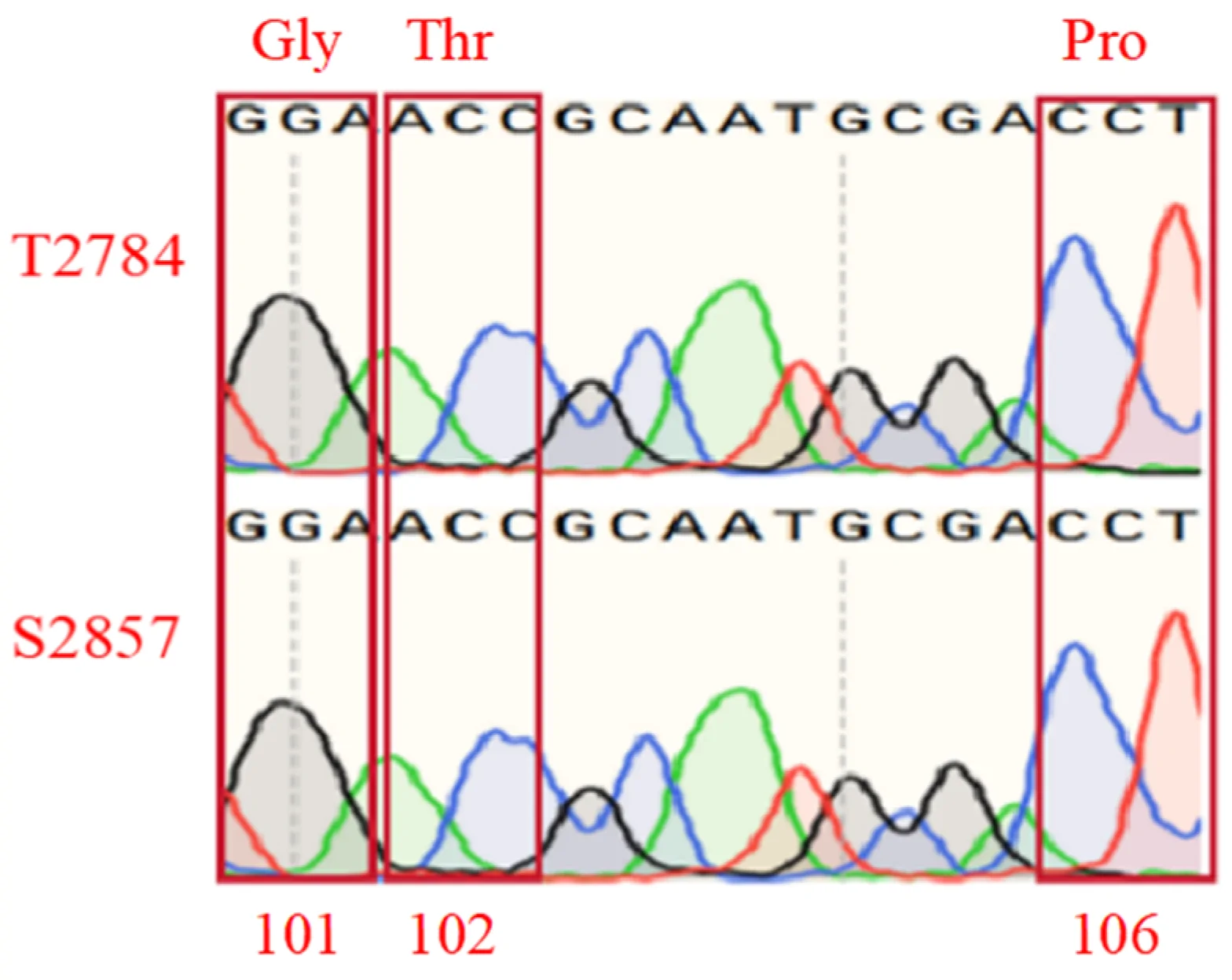
Partial nucleotide sequences of the *EPSPS* gene in T2784 and S2857 *Abutilon theophrasti* populations.

The *EPSPS* gene sequence was successfully amplified using the primers reported by Jia Fang [23]. In the chromatogram, the four colored peaks represent four different dideoxynucleotides. Sequence alignment of the *EPSPS* gene from the T2784 and S2857 populations revealed no nucleotide substitutions resulting in amino acid changes at the three previously reported mutation sites. No amino acid substitutions were detected in any of the 10 sequenced T2784 plants or 10 sequenced S2857 plants. Furthermore, no novel amino acid substitution sites were identified in the amplified *EPSPS* sequence. Because the entire *EPSPS* locus was not sequenced, these results do not exclude mutations in unamplified regions, structural variation, or sequence variation among additional *EPSPS* copies (Figure 2).

### 2.3. Relative Expression Level of the EPSPS Gene

**Figure 3:**
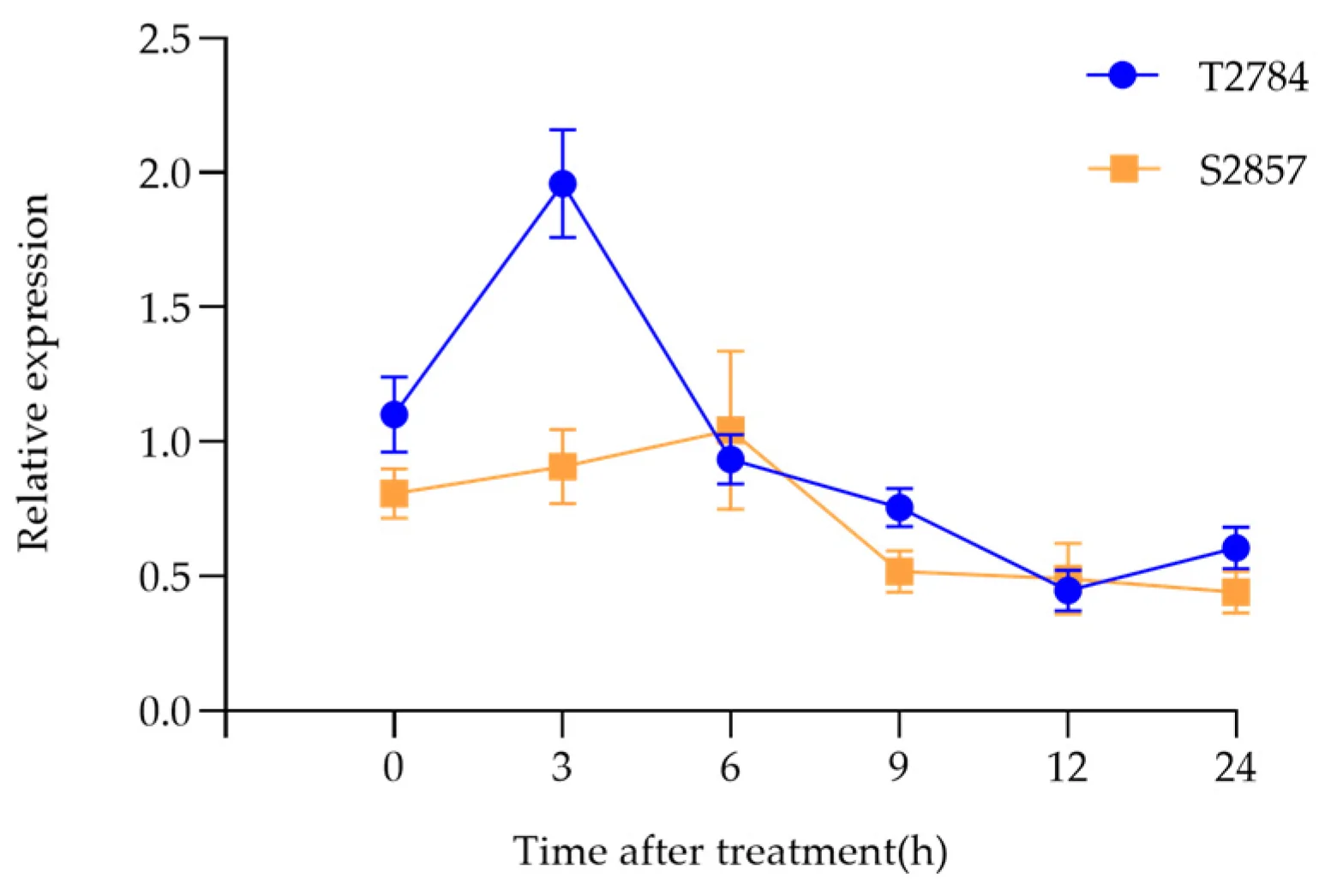
Relative expression levels of the *EPSPS* gene in the T2784 and S2857 *Abutilon theophrasti* populations following glyphosate treatment. Error bars represent the SD among biological replicates.

In the absence of glyphosate treatment, the *EPSPS* transcript level in T2784 was 1.35-fold that in S2857. Following treatment with 1125 g a.i. ha^−1^ glyphosate, *EPSPS* expression in both populations initially increased and subsequently decreased. The maximum expression level occurred at 3 h after treatment in T2784 and at 6 h after treatment in S2857. The maximum relative expression levels were 1.818 and 0.834 in T2784 and S2857, respectively. However, none of the between-population comparisons, including the comparison at 3 h, reached statistical significance (*p* ≥ 0.05). These results indicate that *EPSPS* transcript abundance did not differ significantly between the two populations at the sampled time points, including the 3 h time point. Because no correlation analysis was performed, these data do not establish a statistical correlation between *EPSPS* transcript abundance and glyphosate tolerance (Figure 3).

### 2.4. Effect of Glyphosate on EPSPS Activity In Vitro

**Figure 4:**
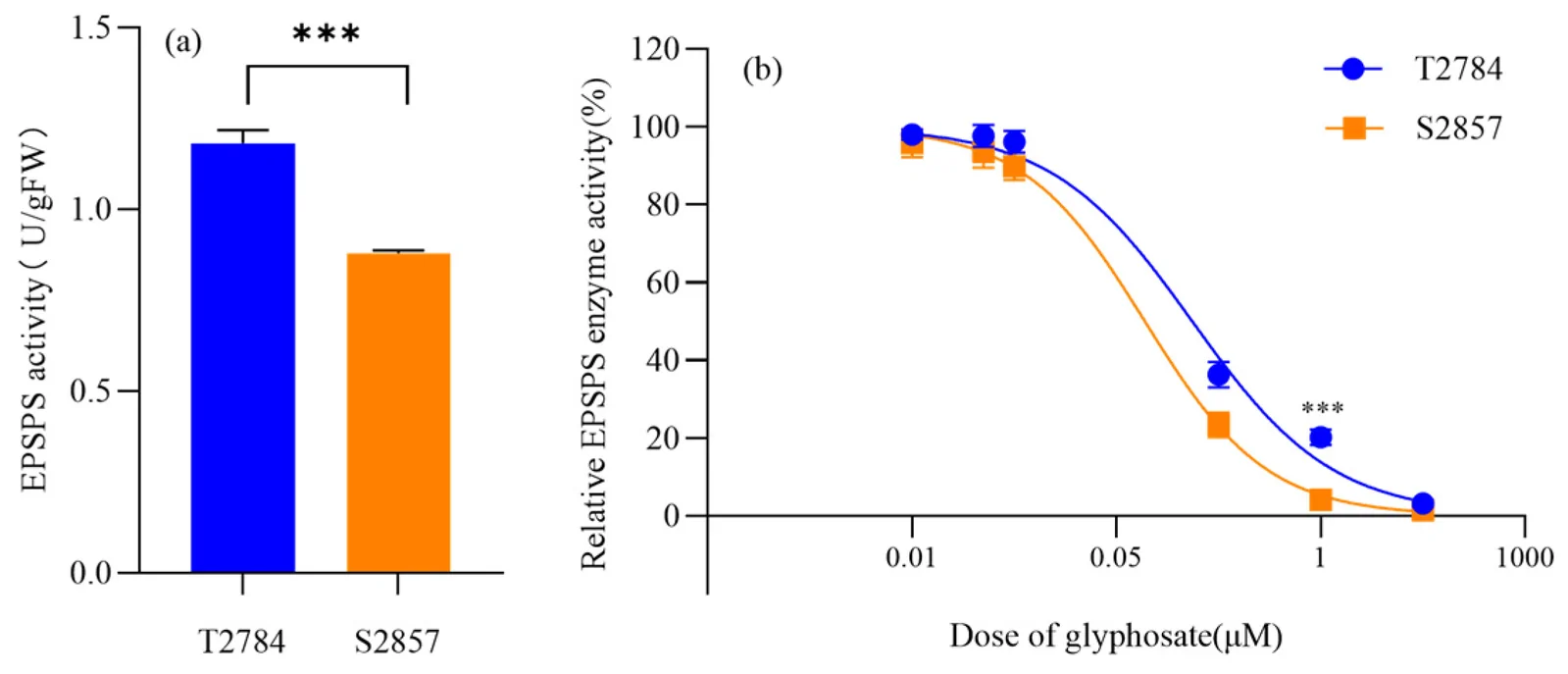
In vitro EPSPS activity in the absence of glyphosate (**a**) and the response of EPSPS activity to glyphosate (**b**) in *Abutilon theophrasti*. Data are presented as means ± standard deviation. *** indicates significant differences according to Student’s *t*-test at *p* < 0.001, respectively.

In the absence of glyphosate, EPSPS activity was 1.1815 U g^−1^ fresh weight in T2784 and 0.8733 U g^−1^ fresh weight in S2857 (Figure 4a). EPSPS activity in T2784 was significantly higher than that in S2857 (*p* < 0.001). Following the addition of glyphosate, EPSPS activity in both populations decreased with increasing glyphosate concentration (Figure 4b). The IC_50_ value for glyphosate inhibition of EPSPS activity was 0.2794 μmol L^−1^ in S2857 and 0.7431 μmol L^−1^ in T2784. Thus, the IC_50_ value in T2784 was 2.66-fold higher than that in S2857.

### 2.5. Glyphosate Absorption and Apparent Disappearance of Parent Glyphosate

**Figure 5:**
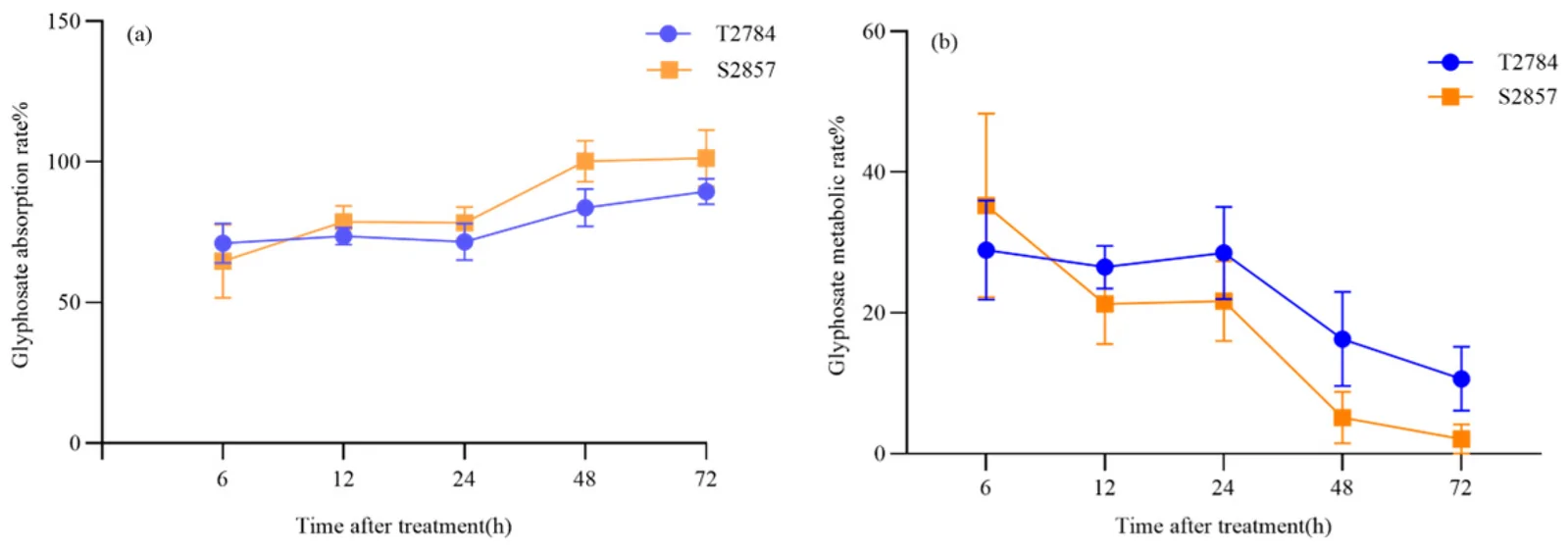
Glyphosate absorption (**a**) and apparent disappearance of recoverable parent glyphosate from the treated leaf (**b**) in the T2784 and S2857 *Abutilon theophrasti* populations. Panel (**b**) does not represent a direct measurement of glyphosate metabolism.

The calibration curve for glyphosate ion peak area versus glyphosate concentration showed good linearity, with an R^2^ value greater than 0.99. In the spike-recovery experiments, recoveries at 0.1, 0.5, and 1.0 mg kg^−1^ were 77.13%, 84.76%, and 89.98%, respectively. These values met the requirements of pesticide residue analysis standards, confirming the reliability of the analytical method. Glyphosate absorption in both T2784 and S2857 increased over time after treatment (Figure 5a). At all sampling times (6–72 h), no significant difference in glyphosate absorption was detected between the two populations. The calculated apparent disappearance of recoverable parent glyphosate from the treated leaf increased over the sampling period in both populations (Figure 5b). At 6 h after treatment, this value was numerically higher in S2857 than in T2784; however, no significant between-population difference was detected at any sampling time. Because glyphosate metabolites and glyphosate in other plant tissues were not quantified, these data do not measure metabolic conversion.

Because only the parent glyphosate compound was quantified and specific glyphosate metabolites were not measured, the metabolism values reported here represent estimates based on the disappearance of recoverable parent glyphosate. Loss of parent glyphosate may reflect metabolism, movement out of the treated leaf, redistribution within the plant, or other physiological processes. Accordingly, these results do not support enhanced apparent metabolism in T2784, but direct confirmation of glyphosate metabolism would require simultaneous quantification of glyphosate and its metabolites, particularly aminomethylphosphonic acid (AMPA).

### 2.6. Effect of a Metabolic Enzyme Inhibitor on Glyphosate Sensitivity

To determine whether enhanced metabolism contributed to glyphosate tolerance, plants were pretreated with the cytochrome P450 inhibitor malathion. Values are GR_50_ estimates with 95% confidence intervals. The absence of a statistically significant difference indicates that the observed changes from 953.0 to 949.3 g a.i. ha^−1^ in T2784 and from 415.3 to 405.4 g a.i. ha^−1^ in S2857.

Pretreatment with malathion did not significantly alter glyphosate sensitivity in either population. Thus, the present results provide no evidence for a major malathion-sensitive component contributing to the differential glyphosate response under the tested conditions (Table 2).

## 3. Discussion

*Abutilon theophrasti* is a serious broadleaf weed in spring soybean-producing regions of northeastern China. By competing for light, water, and nutrients, it can cause substantial soybean yield losses [9]. Chemical control remains the primary method used to manage this weed. Herbicides such as fomesafen, bentazone, and diclosulam have been investigated for the control of *A. theophrasti* in soybean fields [24,25,26]. Gu [27] investigated the use of glyphosate for controlling the noxious weed *Sonchus arvensis* L. In the present study, mature T2784 seeds were collected from a genetically modified soybean field in Hinggan League, Inner Mongolia Autonomous Region, in 2023. The whole-plant bioassay showed that the T2784 population had a GR_50_ of 953.0 g a.i.ha^−1^ and a tolerance index of 2.29, indicating low-level glyphosate tolerance.

*EPSPS* is the primary target site of glyphosate. Changes in its gene sequence, expression level, or enzyme activity may affect plant sensitivity to glyphosate [28,29]. Three major amino acid substitution sites associated with glyphosate resistance have been reported in *EPSPS*: Gly101Ala, Thr102Ile, and Pro106Ser/Ala/Thr/Leu/Ile. In addition to these classical sites, a Pro533Ser substitution has been identified in a highly glyphosate-resistant *Echinochloa crus-galli* population without *EPSPS* gene amplification. Previous studies have also reported increased *EPSPS* expression after glyphosate treatment in glyphosate-tolerant wild soybean [30]. The P106S substitution confers high glyphosate resistance in *Eleusine indica* [31], whereas the combined T102I, A103V, and P106S substitutions confer extremely high glyphosate resistance in *Amaranthus hybridus* [32].

First, the experiments directly demonstrated that the estimated GR_50_ was 953.0 g a.i. ha^−1^ for T2784 and 415.3 g a.i. ha^−1^ for S2857. The tolerance index was 2.29, and T2784 had a higher whole-plant GR_50_ than S2857. No amino acid substitutions were detected within the amplified *EPSPS* region in the analyzed plants, *EPSPS* transcript abundance did not differ significantly between the populations at the sampled time points, and EPSPS activity in T2784 was 2.66-fold higher than that in S2857 under the in vitro assay conditions. The experiments also showed no significant population differences in glyphosate absorption or in the estimated apparent disappearance of recoverable parent glyphosate from the treated leaf. Furthermore, malathion pretreatment did not significantly alter GR_50_ in either population under the tested conditions.

Second, the higher measured EPSPS activity and higher in vitro IC_50_ in T2784 were associated with its lower whole-plant glyphosate sensitivity. Because EPSPS activity was expressed per gram fresh weight, the observed difference may reflect differences in EPSPS protein abundance, total soluble protein content, extract composition, or intrinsic enzyme properties. EPSPS protein abundance, total-protein-normalized activity, enzyme purity, and purified-enzyme kinetics were not determined. Therefore, the similarity between the ratios does not show that the enzyme assay difference quantitatively explains the whole-plant response.

Third, several mechanisms remain unresolved. Because *EPSPS* genomic copy number was not measured, the present study does not exclude *EPSPS* gene amplification. In addition, because only an amplified *EPSPS* region was sequenced, mutations in unamplified regions, additional *EPSPS* copies, or regulatory regions outside the analyzed fragment were not excluded. The estimated apparent disappearance of recoverable parent glyphosate from the treated leaf also does not directly demonstrate glyphosate metabolism or provide a whole-plant mass balance. The observed disappearance may reflect metabolism, translocation to other tissues, redistribution, sequestration, or incomplete analytical recovery. Specific glyphosate metabolites and glyphosate concentrations in untreated leaves, stems, shoot apices, and roots were not determined.

ABC transporters, glutathione S-transferases, glycosyltransferases, aldo-keto reductases, and other oxidoreductases were not measured in the present study. Their possible involvement should therefore be considered a hypothesis for future investigation rather than a demonstrated explanation for the T2784 phenotype. Future studies should use targeted molecular, biochemical, and physiological assays to evaluate these possibilities, including transcript and protein abundance measurements, enzyme-activity assays, transporter-function analyses, and quantification of glyphosate and its metabolites across major plant tissues. The contribution of individual CYP450 enzymes also remains unresolved because malathion is an indirect and non-specific diagnostic inhibitor.

Further studies should quantify glyphosate separately in leaf-surface washes, treated leaves, untreated leaves, stems, shoot apices, and roots to establish a complete glyphosate mass balance. Simultaneous determination of glyphosate and AMPA would be required to confirm whether metabolism differs between the populations. EPSPS protein abundance and the kinetic properties of purified EPSPS enzymes should also be measured to determine the biochemical basis of the higher EPSPS activity in T2784. Transcriptomic and proteomic comparisons could further identify candidate genes associated with membrane transport, vacuolar sequestration, xenobiotic metabolism, and oxidative-stress tolerance.

From a management perspective, the detection of low-level glyphosate tolerance indicates that reliance on glyphosate alone may increase the survival and subsequent selection of less-sensitive *A. theophrasti* individuals. Integrated weed management should therefore combine effective alternative herbicide modes of action with agronomic control practices. In terms of chemical control, the 33% Quinclorac·bentazone emulsifiable concentrate has been reported to effectively control annual weeds, including *A. theophrasti*, in soybean fields at application rates above 1500 mL ha^−1^. At 30 days after treatment, total plant control and fresh-weight control reached 85.5–97.7% and 89.3–99.2%, respectively, without apparent damage to soybean [26]. In terms of agronomic management, soybean planted at narrow row spacing can suppress *A. theophrasti* growth, leaf area development, and seed production more effectively than soybean planted at wide row spacing. In addition, delayed emergence of *A. theophrasti* reduces its growth and reproductive capacity. These findings support integrated management strategies combining narrow-row soybean production with early control of *A. theophrasti* [33].

## 4. Materials and Methods

### 4.1. Plant Materials and Growth Conditions

Seeds of a putatively glyphosate-tolerant *A. theophrasti* population (T2784) were collected from a genetically modified soybean field in Hinggan League, Inner Mongolia Autonomous Region, China (N45°01′, E 121°30′), in 2023. Seeds of the susceptible population S2857 were collected from a field with no history of herbicide use in Tongliao, Inner Mongolia, China (N43°45′, E121°19′). After natural air-drying, the seeds were stored in a seed bank. To enhance germination, seeds were soaked in water at 45 °C for 30 min and then air-dried. Approximately 20 seeds were sown in each 9 cm × 9 cm plastic pot. Seeds were covered with approximately 1 cm of soil, and soil moisture was maintained by placing the pots in trays containing water. At 7 days after sowing, seedlings were thinned to six uniformly sized plants per pot. Seedlings were cultivated in a controlled greenhouse environment with day/night temperatures of 30 ± 3 °C (16 h light) and 25 ± 3 °C (8 h dark), with sufficient water and nutrients to ensure healthy growth.

This growth stage was selected for herbicide application because it represents an early, actively growing period in the plant’s development, which enhances the accuracy and reproducibility of herbicide tolerance assessments due to increased sensitivity to treatment.

### 4.2. Whole-Plant Glyphosate Sensitivity Experiment

Herbicide tolerance was assessed through dose–response assays using a laboratory sprayer (3WP-2000, Nanjing Agricultural Mechanization Research Institute, Nanjing, China) equipped with a flat-fan nozzle delivering 450 L ha^−1^. When plants reached the 5–6 leaf stage, they were treated with glyphosate using a 3WP-2000 spray tower at a spray volume of 450 L ha^−1^. Glyphosate (41% isopropylamine salt, Monsanto Company, Lindbergh Boulevard, St. Louis, MO, USA.) doses (g a.i. ha^−1^) were set as follows: 0 (control), 140.625, 281.25, 562.5, 1125, and 2250. At 21 days after treatment, aboveground fresh weight was measured. The experiment followed a completely randomized design with three biological replicates (pots) per herbicide dose. Each treatment was repeated in two independent experimental runs to ensure reproducibility.

### 4.3. EPSPS Gene Sequence Analysis

Ten untreated plants were randomly selected from each population. Genomic DNA extraction was performed using a commercial DNA extraction kit (Tiangen Biochemical, Beijing, China). Primers were designed using SnapGene based on the previously reported *A. theophrasti EPSPS* cDNA sequence. The primer information is provided in Table 3.

PCR amplification was conducted in a 20 μL reaction mixture containing 1 μL of gDNA template at 50 ng μL^−1^, 0.5 μL of each primer at 10 μmol L^−1^, 10 μL of 2×Taq PCR Master Mix, and 8 μL of nuclease-free water. The PCR program consisted of an initial denaturation at 95 °C for 2 min, followed by 34 cycles of 95 °C for 15 s, 60 °C for 15 s, and 72 °C for 1 min, and a final extension at 72 °C for 5 min. PCR products were separated on 1% agarose gels. Products of the expected size were purified and bidirectionally sequenced. Sequence alignment was performed using SnapGene 6.0.2.

### 4.4. EPSPS Gene Expression Analysis

Plants at the 5–6 leaf stage were treated with glyphosate at 1125 g a.i. ha^−1^. Leaf tissue was collected at 0, 3, 6, 9, 12, and 24 h after treatment. Total RNA was extracted using the TransZol Up Plus RNA Kit (TransGen Biotech, Beijing, China). RNA integrity was assessed by 1% agarose gel electrophoresis, RNA concentrations were measured with a NanoDrop spectrophotometer (Thermo Scientific, Waltham, MA, USA), and first-strand cDNA was synthesized using a reverse-transcription system (TransGen Biotech, Beijing, China).

qRT-PCR was conducted on an ABI 7500 Real-Time PCR System (Applied Biosystems, Foster City, CA, USA). Each 20 μL reaction contained 10 μL of 2×TransStart^®^ Top Green qPCR SuperMix (TransGen Biotech), 0.4 μL of each primer, 1 μL of cDNA template, and 8.2 μL of RNase-free ddH_2_O. The amplification program was 95 °C for 2 min, followed by 45 cycles of 95 °C for 10 s, 60 °C for 15 s, and 72 °C for 20 s. Melting curve analysis was conducted at the end of amplification to verify primer specificity. Three biological replicates and two technical replicates were included for each sample.

Relative expression levels of the target gene were calculated using the 2^−ΔΔCt^ method, with *ACTIN* as the endogenous reference gene for normalization. Three biological replicates and two technical replicates were included for each sample.

### 4.5. In Vitro EPSPS Activity Assay

Leaves from untreated plants at the 5–6 leaf stage were collected. For each population, 2 g of leaf tissue was ground thoroughly in liquid nitrogen and homogenized in 18 mL of PBS buffer. The homogenate was centrifuged at 5000× *g* for 5–10 min in 4 °C, and the supernatant was collected for analysis.

EPSPS enzyme activity was assayed in a reaction mixture containing 50 mM MOPS-KOH (pH 7.2), 0.5 mM MgCl_2_, 15% (*v*/*v*) glycerol, 1.5 mM inosine, 0.05 mM Amplex Red, 0.2 U/mL nucleoside phosphorylase, 0.4 U/mL xanthine oxidase, 1.0 U/mL horseradish peroxidase, and varying concentrations of phosphoenolpyruvate (PEP), shikimate 3-phosphate (S3P), and glyphosate. The assay was carried out in a 96-well plate with a final volume of 50 µL. Each concentration included three replicates, and the experiment was conducted twice.

### 4.6. Glyphosate Absorption and Metabolism

At the 5-leaf stage, 10 μL of glyphosate solution at 8 g a.i. L^−1^ was evenly applied to the upper surface of the third leaf using a microsyringe, corresponding to a nominal application amount of 80 μg glyphosate per treated leaf. This localized micro-application format was selected to deliver a defined and reproducible amount of glyphosate to a single leaf for absorption and parent-compound recovery measurements. Each treatment included three replicates, and the experiment was repeated three times. Samples were collected at 6, 12, 24, 48, and 72 h after treatment.

At each sampling time, the treated leaf was excised, and its surface was rinsed with a fixed volume of methanol/water (1:1, *v*/*v*) to recover glyphosate that had not entered the leaf tissue. The leaf-surface rinse was collected in a centrifuge tube and retained for the determination of unabsorbed glyphosate. After surface washing, the treated leaf was immediately frozen in liquid nitrogen and stored at −80 °C until extraction.

Leaf tissue was ground into powder in liquid nitrogen. One gram of powder was transferred to a 50 mL centrifuge tube, and 20 mL of methanol/water (1:1, containing 1% formic acid) was added. The samples were shaken for 20 min, sonicated for 5 min, and centrifuged at 10,000 rpm for 15 min. Subsequent experimental procedures were carried out following the method described by Paiva [34].

The absorption and metabolism of glyphosate in *A. theophrasti* were determined using a QTRAP 5500 triple-quadrupole linear ion-trap mass spectrometer (AB Sciex, Framingham, MA, USA). The chromatographic column was a Metrosep A Supp 5 column (150 mm × 4.0 mm, 5 μm; Metrohm, Herisau, Switzerland). The mobile phases were ultrapure water (A) and 200 mmol L^−1^ ammonium bicarbonate containing 0.1% ammonia water (B). The flow rate was 0.6 mL min^−1^, the column temperature was 40 °C, and the injection volume was 5 μL. The gradient program was as follows: 0–0.5 min, 10% B; 0.5–1.0 min, 10–40% B; 1.0–4.0 min, 40–80% B; 4.0–5.0 min, 80–82.5% B; 5.0–6.0 min, 82.5–95% B; 6.0–7.0 min, 95% B; 7.0–7.1 min, 95–10% B; and 7.1–10.0 min, 10% B.

### 4.7. Effect of Malathion Pretreatment on Glyphosate Sensitivity

To determine whether CYP450 monooxygenases contributed to glyphosate tolerance in T2784, plants were pretreated with malathion before glyphosate application. A preliminary experiment showed that malathion at 1000 g ha^−1^ did not affect the growth of either population. Therefore, this concentration was used in the subsequent experiment.

Plants at the 5–6 leaf stage were sprayed with malathion at 1000 g ha^−1^. One hour later, glyphosate was applied using the same method and dose range described in Section 4.2. At 21 days after treatment, aboveground fresh weight was measured and the GR_50_ values were calculated. The experiment comprised two independent repetitions, with three pots per treatment and one pot representing one replicate.

### 4.8. Data Analysis

Data were initially processed using Microsoft Excel. Dose–response analyses were performed using SigmaPlot 14.0 (SigmaPlot Software, Chicago, IL, USA). The log-logistic model was applied to calculate the herbicide dose required to reduce plant biomass by 50% (GR_50_) and the EPSPS-inhibitor concentration needed to inhibit 50% of enzyme activity (IC_50_):

$$y\;=\;C\;+\;\frac{\left(D\;-\;C\right)}{1\;+\;({\frac{x}{{\mathrm{GR}}_{50}})}^{b}}$$

where x is the glyphosate dose, y is the fresh weight of treated plants expressed as a percentage of the untreated control, C and D are the lower and upper asymptotes, respectively, and b is the slope at the GR_50_.

Glyphosate absorption was calculated as follows:

$$\mathrm{Glyphosate}\;\mathrm{absorption}\;\mathrm{rate}\;(\%)\;=\;\frac{\left(A1-A2\right)}{A1}\;\times \;100\%$$

where A1 is the nominal amount of glyphosate applied to the treated leaf and A2 is the amount of unabsorbed glyphosate recovered from the leaf-surface rinse.

The estimated glyphosate metabolism rate was calculated operationally from the disappearance of recoverable parent glyphosate:

$$\mathrm{Estimated}\;\mathrm{apparent}\;\mathrm{loss}\;\mathrm{of}\;\mathrm{parent}\;\mathrm{glyphosate}\;(\%)\;=\;\frac{\left(A3-A4\right)}{A3}\;\times \;100\%$$

where A3 is the amount of glyphosate absorbed by the treated leaf, calculated as A1 − A2, and A4 is the amount of recoverable parent glyphosate extracted from the treated leaf tissue. Because glyphosate metabolites were not directly quantified, this parameter represents the apparent disappearance of parent glyphosate and may reflect metabolism, translocation out of the treated leaf, redistribution within the plant, sequestration, or incomplete recovery. Therefore, it was not interpreted as direct evidence of biochemical glyphosate metabolism.

Graphs were prepared using SigmaPlot 14.0 and GraphPad Prism 10.6. Statistical analyses were conducted using SPSS 27.0. The effects of population, sampling time, and their interaction on glyphosate absorption and estimated metabolism were evaluated using two-way analysis of variance. When significant effects were detected, means were separated using an appropriate multiple-comparison test at *p* < 0.05. Other comparisons between the two populations were performed using Student’s *t*-test. Data are presented as means ± standard deviation.

## 5. Conclusions

Overall, the lower whole-plant glyphosate sensitivity of T2784 was associated with higher EPSPS activity when expressed per gram fresh weight and a higher in vitro IC_50_ for glyphosate inhibition. The present study did not detect amino acid substitutions within the amplified *EPSPS* region, significant differences in *EPSPS* transcript abundance at the sampled time points, differences in glyphosate absorption, or differences in the estimated apparent disappearance of recoverable parent glyphosate from the treated leaf. Because only the parent compound in the treated leaf was quantified, these values represent operational estimates of parent-glyphosate disappearance and do not establish glyphosate metabolism or provide a whole-plant mass balance. Pretreatment with malathion did not significantly alter glyphosate sensitivity in either population. Thus, the present results provide no evidence for a major malathion-sensitive component contributing to the differential glyphosate response under the tested conditions.

In addition, whole-plant translocation and subcellular sequestration were not directly determined. These conclusions apply to the comparison between T2784 and S2857. Further studies should include additional independently collected *A. theophrasti* populations with contrasting glyphosate responses and assess their genetic backgrounds. Measurements of EPSPS protein abundance, enzyme properties, whole-plant glyphosate distribution, and glyphosate metabolites would help determine whether the associations observed here are reproducible and identify their underlying causes.

## Figures and Tables

**Table 1:** Glyphosate doses causing a 50% reduction in aboveground fresh biomass in the S2857 and T2784 populations.

Population	Glyphosate						
	GR_50_^a^ (g a.i. ha^−1^)	TI ^b^	Min	Max	Hillslope	R^2^	Adjust R^2^
S2857	415.3 ± 6.53	−	−9.79	99.13	−1.14	0.99	0.98
T2784	953.0 ± 4.61	2.29	−7.54	99.89	−2.47	0.99	0.99

GR_50_^a^: herbicide concentration that caused a 50% reduction in plant growth. TI ^b^ (tolerance index) = GR_50_ of tolerant population/GR_50_ of susceptible population. Data are mean ± SD.

**Table 2:** Sensitivity of *Abutilon theophrasti* populations T2784 and S2857 to glyphosate following pretreatment with malathion.

Population	GR_50_^a^ (g a.i. ha^−1^)		
	Glyphosate	Malathion + Glyphosate	95%CI
S2857	415.3 ± 6.53	405.4 ± 2.74	604.2–1318
T2784	953.0 ± 4.61	949.3 ± 4.25	254.3–680.6

Data are mean ± SD. GR_50_^a^: herbicide concentration that caused a 50% reduction in plant growth.

**Table 3:** Primers used for amplification of the *EPSPS* gene in *Abutilon theophrasti*.

Primers	Sequence (5′–3′)	Tm (°C)	Purpose of Primers
*EPSPS*-F	TTGAACAGCAGCAATGGTAAGTTGG	59.0	Amplify the *EPSPS*
*EPSPS*-R	GCACAGGCGGCAAGAGAGAAT	58.8	
*EPSPS*-1F	TCGTGTGGACATGACAGTGA	54.9	qRT-PCR analysis of *EPSPS* gene
*EPSPS*-1R	CGGCGGTAATAGCAGCAGTAAG	55.0	
*ACT*-F	AATGGCCGATGGTGAGGA	58.9	qRT-PCR analysis of *Actin* gene
*ACT*-R	TCCTTCTGACCCATCCCAAC	61.6	

## Data Availability

The data presented in this study are available on request from the corresponding authors.

## References

[1] Messina M. (2022). Perspective: Soybeans Can Help Address the Caloric and Protein Needs of a Growing Global Population. Front. Nutr..

[2] Cai H.L., Gui S. (2024). Study on Market Effects and Risk Prevention of Expanding Soybean Planting Policies in Northeast China. Econ. Rev..

[3] Dong S., Gao Y., Xin L., Ding W. (2026). Biological characteristics of transgenic glyphosate-resistant semiwild soybean and its competitive ability against weeds for survival. Sci. Rep..

[4] Luo Y. (2025). Problems and Countermeasures for the Industrialization of Biotech Soybean Breeding in China: Based on Surveys in Some Pilot Areas. Chin. J. Oil Crop Sci..

[5] Sandhu R., Chaudhary N., Shams R., Dash K.K. (2025). Genetically modified crops and sustainable development: Navigating challenges and opportunities. Food Sci. Biotechnol..

[6] Liu L., Zhang L., Fang Z., Shen W., Yin X., Ren Z., Yu Q., Liang J., Liu B. (2024). Glyphosate resistance and no fitness cost in backcross offspring of wild soybean and transgenic soybean with *EPSPS* gene. BMC Plant Biol..

[7] Li L., Pang L., Chen F., Zhang L., Zhou F., Li R., Yan H. (2024). *Abutilon theophrasti* Medic.: A Review of its Traditional Uses, Phytochemistry, Pharmacology, and Pharmacokinetics. Nat. Prod. Commun..

[8] Loddo D., Bozic D., Calha I., Dorado J. (2019). Variability in seedling emergence for European and North American populations of *Abutilon theophrasti*. Weed Res..

[9] Norsworthy J.K., Ward S.M., Shaw D.R., Llewellyn R.S., Nichols R.L., Webster T.M., Bradley K.W., Frisvold G., Powles S.B., Burgos N.R. (2012). Reducing the risks of herbicide resistance: Best management practices and recommendations. Weed Sci..

[10] Shi S., Cheng J.B., Ahmad N., Zhao W., Tian M., Yuan Z., Li C., Zhao C. (2023). Effects of potential allelochemicals in a water extract of *Abutilon theophrasti* Medik. on germination and growth of *Glycine max* L., *Triticum aestivum* L., and *Zea mays* L.. J. Sci. Food Agric..

[11] Begonia G.B., Aldrich R.J., Salisbury C.D. (2010). Soybean yield and yield components as influenced by canopy heights and duration of competition of velvetleaf (*Abutilon theophrasti* Medik.). Weed Res..

[12] Warwick S.I., Black L.D. (1988). The biology of Canadian weeds. 90. *Abutilon theophrasti*. Can. J. Plant Sci..

[13] Baskin J.M., Baskin C.C., Leck M.A., Parker V.T., Simpson R.L. (1989). Physiology of dormancy and germination in relation to seed bank ecology. Ecology of Soil Seed Banks.

[14] Heap I. (2023). The International Survey of Herbicide Resistant Weeds. https://www.weedscience.org.

[15] Duke S.O., Powles S.B. (2008). Glyphosate: A once-in-a-century herbicide. Pest. Manag. Sci..

[16] Smedbol E., Gomes M.P., Paquet S., Labrecque M., Lepage L., Lucotte M., Juneau P. (2018). Effects of low concentrations of glyphosate-based herbicide factor 540^®^ on an agricultural stream freshwater phytoplankton community. Chemosphere.

[17] Heap I. (2014). Global perspective of herbicide-resistant weeds. Pest Manag. Sci..

[18] Benbrook C.M. (2016). Trends in glyphosate herbicide use in the United States and globally. Environ. Sci. Eur..

[19] Han Y.J., Meng Z.W., Wu J., Zhang Y., Zhou F.Y. (2023). Resistance of Echinochloa crus-galli to cyhalofop-butyl and oxadiargyl and its target molecular mechanism in paddy fields of Anhui Province. J. Plant Prot..

[20] Nandula V.K., Reddy K.N., Koger C.H., Poston D.H., Rimando A.M., Duke S.O., Bond J.A., Ribeiro D.N. (2012). Multiple Resistance to Glyphosate and Pyrithiobac in Palmer Amaranth (*Amaranthus palmeri*) from Mississippi and Response to Flumiclorac. Weed Sci..

[21] Délye C. (2013). Unravelling the genetic bases of non-target-site-based resistance (NTSR) to herbicides: A major challenge for weed science in the forthcoming decade. Pest. Manag. Sci..

[22] Okumu M.N., Robbertse P.J., Vorster B.J., Reinhardt C.F. (2022). The Molecular, Morphological and Genetic Characterization of Glyphosate Resistance in Conyza bonariensis from South Africa. Plants.

[23] Jia F. (2020). Study on Tolerance Levels and Mechanisms of Major Broadleaf Weeds in Maize Fields to Glyphosate. Master’s Thesis.

[24] Luo C., Guo X.T., Wang Y., Cong K.Q., Wei X.F., Guo Y.L. (2024). Application Effect of Bispyribac-sodium in Spring Soybean Fields in Northeast China. Plant Prot..

[25] Wang C., Mu J.Z., Liu J.L. (2000). Field Trial on Weed Control Effect of Fluorosulfuron in Soybean Fields. Mod. Agric..

[26] Guo X.T., Wang Y., Luo C. (2024). Weed Control Effect of 33% Quinclorac·Bentazone Emulsion on Annual Weeds in Soybean Fields. Heilongjiang Agric. Sci..

[27] Gu C.B. (2003). Study on the Weed Control Technology of *Sonchus arvensis* L. in Soybean Fields with Glyphosate. Master’s Thesis.

[28] Sammons R.D., Gaines T.A. (2014). Glyphosate resistance: State of knowledge. Pest. Manag. Sci..

[29] Duke S.O. (2018). The history and current satatus of glyphosate. Pest. Manag. Sci..

[30] Wang Y., Zhang K., Wang Y.L., Si Z., Wang B., Qiao Y. (2022). Identification of Glyphosate Tolerance in Wild Soybeans from Jidong and Preliminary Study on Tolerance Mechanism. J. Nucl. Agric. Sci..

[31] Franci J., Lam K.W., Chuah T.S., Cha T.S. (2020). Genetic diversity and in silico evidence of target-site mutation in the *EPSPS* gene in endowing glyphosate resistance in *Eleusine indica* (L.) from Malaysia. Pestic. Biochem. Physiol..

[32] Resende L.S., Reis G.L.D., Netto A.G., Borsato E.F., Penckowski L.H., Alvarenga J.P., Sales T.A., de Castro Ramalho T., Christoffoleti P.J., Chalfun-Junior A. (2025). Alterations in EPSPS enzyme active site: Changes in position and reduced interaction energy contributing to glyphosate resistance in *Amaranthus hybridus*. Plant Physiol. Biochem..

[33] Hock S.M., Knezevic S.Z., Martin A.R., Lindquist J.L. (2005). Influence of soybean row width and velvetleaf emergence time on velvetleaf (*Abutilon theophrasti*). Weed Sci..

[34] Paiva A.C.P., Assis E.C.D., d’Antonino L., de Queiroz M.E.L.R., da Silva A.A. (2024). Alternative Method for Glyphosate Determination in Unroasted Green Coffee Beans by Liquid Chromatography Tandem Mass Spectrometry (LC–MS/MS). J. Agric. Food Chem..

